# *Leptospira* Immunoglobulin-Like Protein B (LigB) Binds to Both the C-Terminal 23 Amino Acids of Fibrinogen αC Domain and Factor XIII: Insight into the Mechanism of LigB-Mediated Blockage of Fibrinogen α Chain Cross-Linking

**DOI:** 10.1371/journal.pntd.0004974

**Published:** 2016-09-13

**Authors:** Ching-Lin Hsieh, Eric Chang, Andrew Tseng, Christopher Ptak, Li-Chen Wu, Chun-Li Su, Sean P. McDonough, Yi-Pin Lin, Yung-Fu Chang

**Affiliations:** 1 Department of Population Medicine and Diagnostic Sciences, College of Veterinary Medicine, Cornell University, Ithaca, New York, United States of America; 2 Department of Biomedical Science, College of Veterinary Medicine, Cornell University, Ithaca, New York, United States of America; 3 Division of Infectious Disease, Wadsworth Center, New York State Department of Health, Albany, New York, United States of America; Instituto Butantan, BRAZIL

## Abstract

The coagulation system provides a primitive but effective defense against hemorrhage. Soluble fibrinogen (Fg) monomers, composed of α, β and γ chains, are recruited to provide structural support for the formation of a hemostatic plug. Fg binds to platelets and is processed into a cross-linked fibrin polymer by the enzymatic clotting factors, thrombin and Factor XIII (FXIII). The newly formed fibrin-platelet clot can act as barrier to protect against pathogens from entering the bloodstream. Further, injuries caused by bacterial infections can be confined to the initial wound site. Many pathogenic bacteria have Fg-binding adhesins that can circumvent the coagulation pathway and allow the bacteria to sidestep containment. Fg expression is upregulated during lung infection providing an attachment surface for bacteria with the ability to produce Fg-binding adhesins. Fg binding by *leptospira* might play a crucial factor in *Leptospira*-associated pulmonary hemorrhage, the main factor contributing to lethality in severe cases of leptospirosis. The 12^th^ domain of *Leptospira* immunoglobulin-like protein B (LigB12), a leptospiral adhesin, interacts with the C-terminus of FgαC (FgαCC). In this study, the binding site for LigB12 was mapped to the final 23 amino acids at the C-terminal end of FgαCC (FgαCC8). The association of FgαCC8 with LigB12 (ELISA, K_D_ = 0.76 μM; SPR, K_D_ = 0.96 μM) was reduced by mutations of both charged residues (R608, R611 and H614 from FgαCC8; D1061 from LigB12) and hydrophobic residues (I613 from FgαCC8; F1054 and A1065 from LigB12). Additionally, LigB12 bound strongly to FXIII and also inhibited fibrin formation, suggesting that LigB can disrupt coagulation by suppressing FXIII activity. Here, the detailed binding mechanism of a leptospiral adhesin to a host hemostatic factor is characterized for the first time and should provide better insight into the pathogenesis of leptospirosis.

## Author Summary

Leptospirosis, caused by pathogenic *Leptospira spp*., has been increasingly recognized as an emerging zoonosis worldwide. In human cases, clinical presentation can vary from a mild flu-like syndrome to severe multi-organ failure including hepatitis, nephritis and occasionally meningitis. Particularly, pulmonary hemorrhage has become one of the major factors leading to fatality. The host coagulation system normally can be activated to confine damage caused by bacteria. However, this spirochete has developed several virulence proteins to manipulate hemostatic factors including fibrinogen (Fg). Previously, we had observed that *Leptospira* immunoglobulin-like protein B (LigB) can bind to Fg and inhibit fibrin clot formation. In this study, the LigB binding site on fibrinogen was fine-mapped. The key amino acids contributing to this strong pathogen-host interaction were also identified. In addition, LigB bound to factor XIII and further interfered with the cross-linking of Fg. For the first time, a potential mechanism of leptospiral adhesin binding to fibrinogen was revealed, which should provide a better understanding of the pathogenesis of leptospirosis.

## Introduction

*Leptospira* spp are pathogenic spirochetes that cause the most widespread zoonotic disease in the world [1,2]. Leptospirosis is reported regularly in tropical nations and is reemerging in the United States [3,4]. Numerous mammalian hosts, including incidental hosts like humans, can be infected by *Leptospira* at sites of exposed mucous membranes or eroded skin. Direct contact with *Leptospira*-contaminated water is the main transmission mechanism for endemic leptospirosis associated with flooded areas and populations suffering poor hygienic measures [5,6]. Once the spirochete invades the vasculature, it rapidly disseminates throughout the body, reaching target organs (e.g. liver, kidneys and lungs) if the host humoral response does not effectively prevent their spread. Symptoms vary widely from a mild flu-like syndrome to multi-organ failure such as hepatic dysfunction, interstitial nephritis and pulmonary hemorrhage, also known as Weil’s disease. If the infected individual does not receive prompt antibiotic and supportive treatment, fatality may result [7,8].

**Fig 1:**
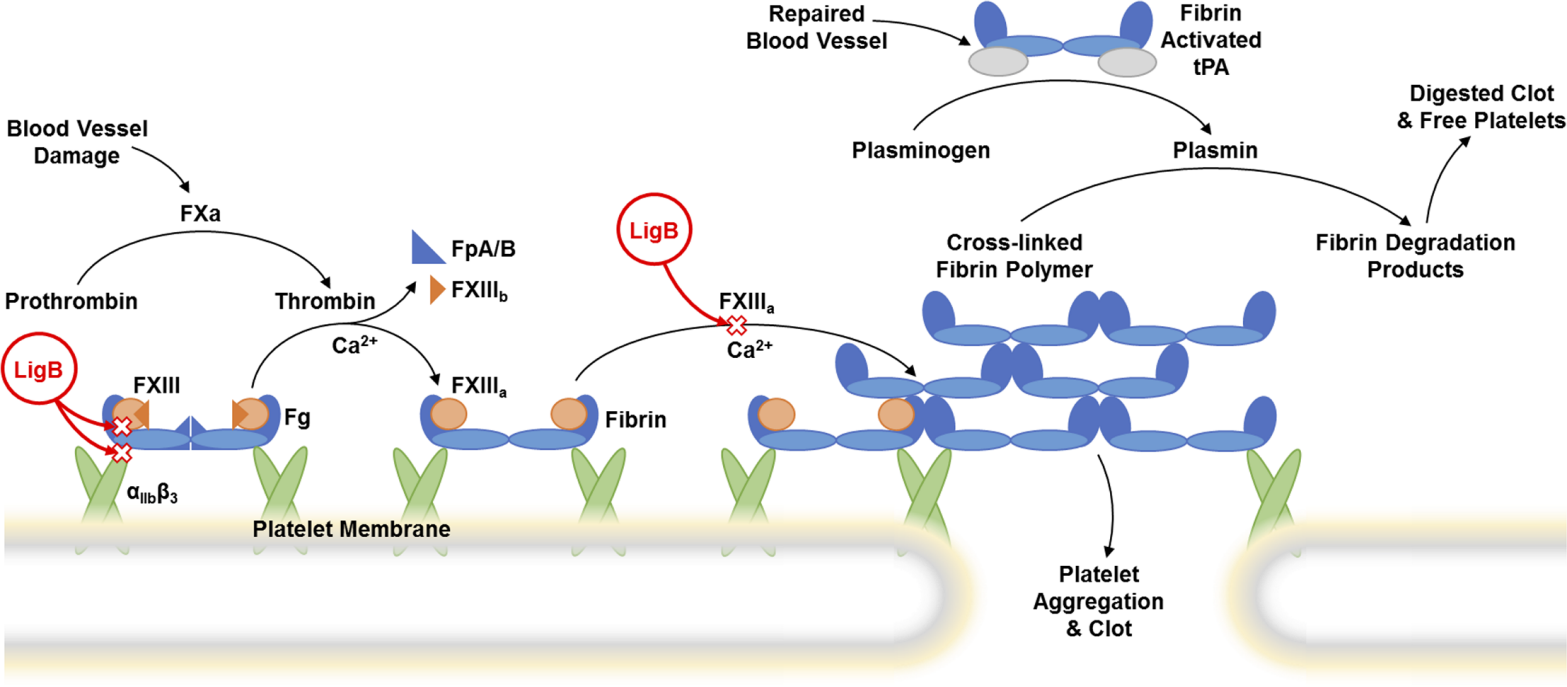
Coagulation cascade with the steps affected by LigB. The coagulation cascade is initiated by blood vessel damage and eventually leads to activation of factor Xa (FXa) which enzymatically processes prothrombin into thrombin. Subsequently, thrombin removal of fibrinopeptides (FpA/B), fibrinopeptide A (FpA) from fibrinogen (Fg) α chain and fibrinopeptide B (FpB) from Fg β chain, yields fibrin. The cross-linking of fibrin polymers is catalyzed by thrombin-activated factor XIII (FXIIIa) followed by the dissociation of subunit FXIIIb. The possible mechanisms of LigB-mediated disruption of the coagulation pathway are indicated by red (**×**)s. LigB inhibition of Fg binding to platelet integrin α_IIb_β3 would block platelet aggregation. In addition, LigB interference with the FXIII-Fg interaction would block FXIII-mediated cross-linking of Fg; thus reducing fibrin clot formation. LigB has not been shown to affect the fibrin degradation pathway where tissue plasminogen activator (tPA) and plasmin play a role.

Fibrinogen (Fg), a 340 kDa plasma glycoprotein, plays a critical role in the coagulation cascade and platelet aggregation (Fig 1). The coagulation pathway can be initiated by disruption of the endothelial lining due to damage by invading pathogens such as *Leptospira* [9]. Subsequently, the clotting factors enzymatically activate in a specific sequence, which finally leads to thrombin activation. Thrombin then proteolytically processes the N-terminal Fg α and β chains, resulting in the release of fibrinopeptide A and B (FpA and FpB) and the exposure of the binding sites for the C-terminal Fg β chain (βC domain) and γ chain (γC domain). Along with the interaction between the N-terminus of Fg and the C-terminus of neighboring Fg, αC domains of Fg associate with each other intermolecularly to promote the lateral aggregation of protofibrils. Eventually, Factor XIII (FXIII) cross-links the α and γ chains of Fg, solidifying the fibrin clot to restrict hemorrhage. Furthermore, the tight adhesion of fibrin to platelets can potentially constrain the spread of the pathogens [10]. However, the critical roles of Fg in coagulation, platelet activation, tissue regeneration and immune responses make it a perfect target for many pathogens [11,12].

To promote systemic infection, hematogenous bacteria, including *Leptospira*, have evolved a variety of surface proteins to cope with host coagulation and immune systems. For example, clumping factor A (ClfA) from *Staphylococcus aureus* inhibits platelet aggregation and fibrin clot formation [13,14]. Serine-aspartate repeat protein G (SdrG) from *S*. *epidermidis* also interferes with the thrombin-mediated coagulation pathway [15]. Several leptospiral adhesins bind to fibrinogen (Fg) and other molecules involved in clotting [16–22]. However, only a few of them can inhibit thrombin-mediated fibrin formation *in vitro* [23]. *Leptospira* immunoglobulin-like (Lig) protein B (LigB) is the only leptospiral adhesin that can block fibrin clot formation and also diminish platelet adhesion and aggregation [17,20]. Previous studies have demonstrated that the Fg binding region is located at the C-terminal Ig-like domains of LigB [17,20]. The Fg-LigB interaction suggests that LigB might play a role in leptospirosis-associated pulmonary hemorrhage, which is a primary factor contributing to lethality in humans [24,25].

Previously, we utilized multifunctional LigB12 to search for potential binding sites in Fg and found that LigB12 binds to the C-terminal region of the FgαC domain (FgαCC) [17]. The binding of LigB to Fg can block fibrin clot formation and stifle platelet aggregation [17]. Importantly, the FgαCC domain serves as a multifaceted receptor interacting with FXIII, integrin α_IIb_β3, plasminogen (PLG), tissue plaminogen activator (tPA), and even itself to maintain normal physiological processes [26]. Here, we fine-map the LigB-binding sites on FgαCC and the Fg-binding region on LigB12. Based on secondary structure prediction and potential functionality of specific regions on FgαCC, we designed and expressed a set of constructs, FgαCC1 (392–426), FgαCC2 (426–507) and FgαCC3 (507–625). ELISA-based binding experiments showed that FgαCC3 retained the same LigB12-binding ability as full-length FgαCC. Additional truncations were generated to identify a minimal binding site, FgαCC8, a 23 amino acid fragment positioned at the C-terminus of FgαCC. The binding of LigB12 to FgαCC8 (K_D_ = 0.959 μM) was mediated by electrostatic and hydrophobic interactions. Amino acids R608, R611, I613 and H614 of FgαCC8 and F1054, D1061 and A1065 of LigB12 all played roles in the LigB12-FgαCC8 binding interface. Furthermore, LigB12 interfered with the FXIII-Fg interaction and inhibited FXIII-assisted cross-linking of Fg α chains.

## Methods

### Bacterial strains and reagents

*Escherichia coli* TOP10 and Rosetta (DE3) strains (Invitrogen and Novagen) were cultured in Luria-Bertani broth (LB) with appropriate antibiotics at 37°C. MaxiSorp™ microtiter plates from NUNC were used for ELISA. Rabbit anti-GST IgG antibody conjugated with horseradish peroxidase (HRP) and rabbit anti-Sumo tag IgG antibody were purchased from GenScript (Piscataway, NJ). Mouse anti-His tag monoclonal antibody was obtained from Invitrogen (Waltham, MA). Peroxidase substrate 3,3’,5,5’- Tetramethylbenzidine (TMB) and solution were purchased from Kirkegaard & Perry Laboratories (Gaithersburg, MD). Biacore CM5 chips and amine coupling kit containing 1-ethyl-3-(3 dimethylaminopropyl) carbodiimide hydrochloride (EDC), N-hydroxysuccinimide (NHS) and ethanolamine-HCl were obtained from GE Healthcare (Pittsburgh, PA). Thrombin and FXIII were purchased from Haematologic Technologies, Inc. (Essex Junction, VT). Human plasma Fg was purchased from EMD Millipore (Billerica, MA).

### Plasmid construction

**Fig 2:**
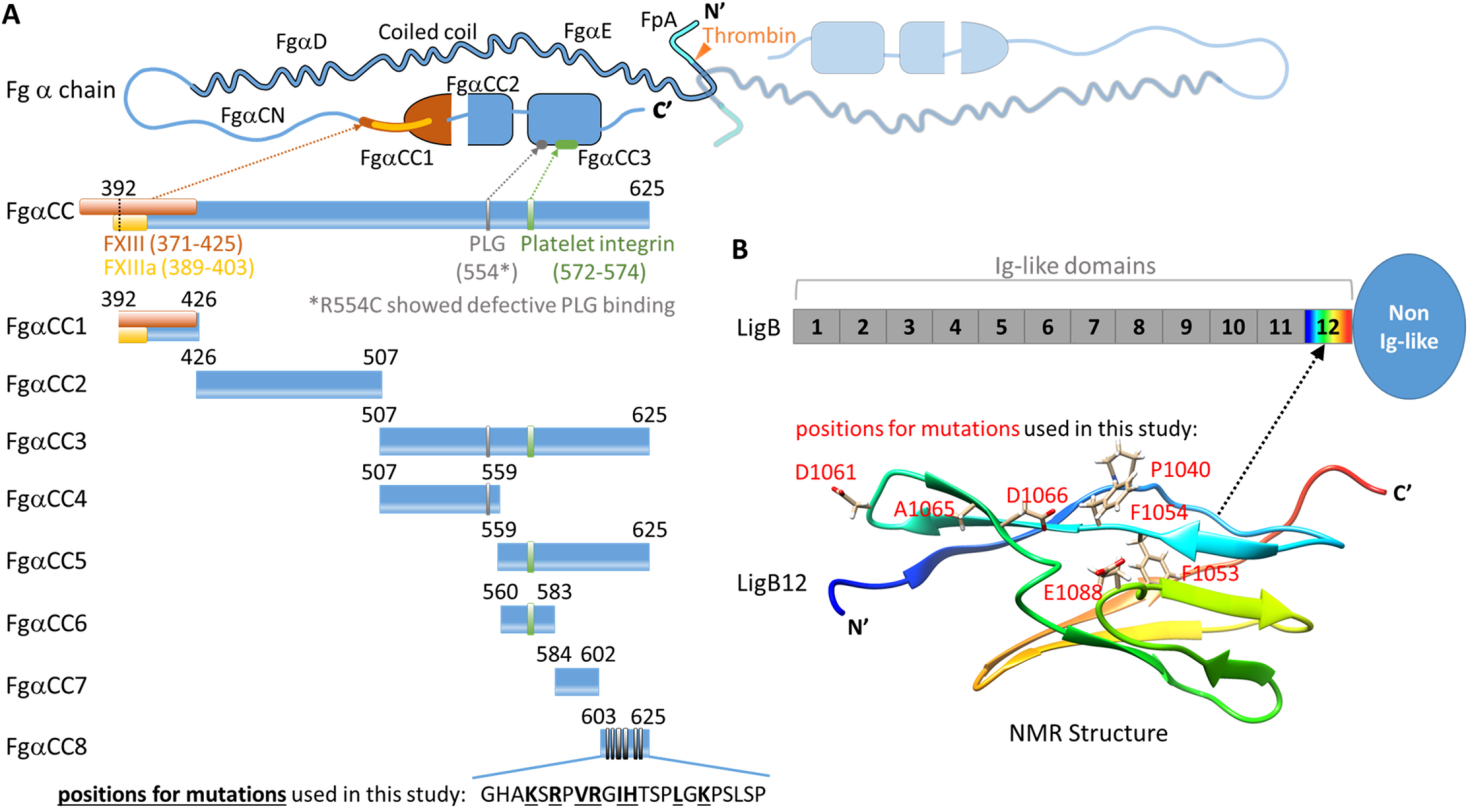
Schematic representation of Fg α chain and LigB protein. **(A)** The dimeric Fg α chain (composed of a labeled monomer and a faded monomer) is composed of N-terminal fibrinopeptide A (FpA), central coiled coil (FgαD and FgαE), and C-terminal domain (FgαC). The thrombin cleavage site responsible for FpA removal is indicated by an orange arrow. FgαC is further divided into an unstructured N-terminal connector (FgαCN) and a partially folded C-terminal domain (FgαCC). All FgαCC truncated constructs used in this study are shown as blue bars with starting and ending residues numbered relative to the mature Fg α chain. The sites for physiological Fg-binding partners (FXIII, FXIIIa, plasminogen (PLG), and platelet integrin) are indicated as color boxes and labeled on FgαCC. The residues of FgαCC8 replaced by alanine for this study are bolded and underlined. **(B)** The LigB bar depicts the twelve LigB Ig-like domains. The non Ig-like domain region (oval) extends from the terminal Ig-like domain (LigB12). The NMR structure of LigB12 is shown as a rainbow ribbon along with the highlighted residues that were mutated in this study.

LigB12 (amino acids 1047–1119 in LigB) was amplified based on the DNA sequences derived from GenBank (*L*. *interrogans* serovar Pomona, GenBank number: FJ030916) and cloned into pET28-SUMO or pGEX-4T-2 vector (GE Healthcare) for expression as His-Sumo tagged or GST-tagged proteins [27]. LigB4 (amino acids 307–403 in LigB) was also constructed and expressed in the same way. All constructs of FgαCC truncations were amplified based on the DNA sequence from human fibrinogen alpha chain (GenBank number: NM_021871.3). FgαCC (amino acids 392–625 in Fg α chain) and its various truncates (Fig 2), including FgαCC1 (amino acids 392–426 in Fg α chain), FgαCC2 (amino acids 426–507 in Fg α chain), FgαCC3 (amino acids 507–625 in Fg α chain), FgαCC4 (amino acids 507–559 in Fg α chain), FgαCC5 (amino acids 560–625 in Fg α chain), FgαCC6 (amino acids 560–583 in Fg α chain), FgαCC7 (amino acids 584–602 in Fg α chain) and FgαCC8 (amino acids 603–625 in Fg α chain) were amplified by PCR using the primers listed in Table 1 and the construct FgαCC/pGEX-4T-2 as a template [17]. All amplified FgαCC fragments were digested with BamHI and HindIII (Invitrogen), and then ligated into pET28-SUMO vector cut with the same pair of restriction enzymes. FgαCC8 was ligated into pET-THGT vector as well in order to express it as a GST tagged protein [28]. For generating FgαCC8 mutants (K606A, R608A, V610A, R611A, I613A, H614A, L618A, K620A), the corresponding primers (Table 1) were utilized to make site-directed mutagenesis with wild-type FgαCC8 /pET28-SUMO serving as the template. As for LigB12 mutants (F1054A, D1061N, A1065K, D1066A and E1088A), the primers used for making site-directed mutagenesis are also listed in Table 1 and wild-type LigB12/pET28-SUMO was used as a template. Finally, LigB12, LigB12 mutants, wild-type FgαCC truncates and FgαCC8 mutants were all subjected to DNA sequencing to exclude any clone with undesired mutations. The sequence-confirmed constructs were then respectively transformed into *E*. *coli* Rosetta strains for protein expression.

**Table 1:** Oligonucleotides used in this study (restriction enzyme sites are underlined; the mutagenesis sites for site-directed mutagenesis primers are also underlined.)

Primer name	Sequence (5’ ➔ 3’)
FgαCC1 fp	CGCGGATCCGGCACATTTGAAGAGGTG
FgαCC1 rp	CCCAAGCTTCTAACCAGTCCTGAGCTC
FgαCC2 fp	CGCGGATCCGGTAAAGAGAAGGTC
FgαCC2 rp	CCCAAGCTTCTATCCAGTTGAGGCAGT
FgαCC3 fp	CGCGGATCCGGAAAAACATTCCCA
FgαCC3 rp	CCCAAGCTTCTAGGGGGACAGGGAAG
FgαCC4 fp	Same as FgαCC3 fp
FgαCC4 rp	CCCAAGCTTCTAACTTGAAGATTTACCACG
FgαCC5 fp	CGCGGATCCTACAGCAAACAATTTACT
FgαCC5 rp	Same as FgαCC3 rp
FgαCC6 fp	Same as FgαCC5 fp
FgαCC6 rp	CCCAAGCTTCTA TTTATAGCTCTTGCTTTC
FgαCC7 fp	CGCGGATCCATGGCAGATGAGGCC
FgαCC7 rp	CCCAAGCTTCTATCTCTTGGTGCTATG
FgαCC8 fp	CGCGGATCCGGGCATGCTAAATCT
FgαCC8 rp	Same as FgαCC3 rp
LigB4 fp	CGGAATTCACTCCAGCAGCCTTA
LigB4 rp	CCGCTCGAGCTACAAAGCAGCTTGTGTAAC
LigB12 fp	CGCGGATCCGCAGCAACCCTTTCT
LigB12 rp	CCCAAGCTTCTACGTGTCCGTTTTGTT
FgαCC8 K606A fp	GGATCCGGGCATGCTGCATCTCGCCCTGTCAGA
FgαCC8 K606A rp	TCTGACAGGGCGAGATGCAGCATGCCCGGATCC
FgαCC8 R608A fp	GGGCATGCTAAATCTGCCCCTGTCAGAGGTATC
FgαCC8 R608A rp	GATACCTCTGACAGGGGCAGATTTAGCATG CCC
FgαCC8 V610A fp	GCTAAATCTCGCCCTGCCAGAGGTATCCACACTTC
FgαCC8 V610A rp	GAAGTGTGGATACCTCTGGCAGGGCGAGATTTAGC
FgαCC8 R611A fp	AAATCTCGCCCTGTCGCAGGTATCCACACTTC
FgαCC8 R611A rp	GAAGTGTGGATACCTGCGACAGGGCGAGATTT
FgαCC8 I613A fp	CGCCCTGTCAGAGGTGCCCACACTTCTCCTTTG
FgαCC8 I613A rp	CAAAGGAGAAGTGTGGGCACCTCTGACAGGGCG
FgαCC8 H614A fp	CCTGTCAGAGGTATCGCCACTTCTCCTTTGGGG
FgαCC8 H614A rp	CCCCAAAGGAGAAGTGGCGATACCTCTGACAGG
FgαCC8 L618A fp	GTATCCACACTTCTCCTGCGGGGAAGCCTTCCCTG
FgαCC8 L618A rp	CAGGGAAGGCTTCCCCGCAGGAGAAGTGTGGATAC
FgαCC8 K620A fp	CTTCTCCTTTGGGGGCGCCTTCCCTGTCCC
FgαCC8 K620A rp	GGGACAGGGAAGGCGCCCCCAAAGGAGAAG
LigB12 F1054A fp	CCGTATCAAAACAATTCGCCGCGGTGGGAACGTATT
LigB12 F1054A rp	AATACGTTCCCACCGCGGCGAATTGTTTTGATACGG
LigB12 D1061N fp	GTGGGAACGTATTCGAATGGAACCAAAGCGGAT
LigB12 D1061N rp	ATCCGCTTTGGTTCCATTCGAATACGTTCCCAC
LigB12 A1065K fp	TCGGATGGAACCAAAAAGGATTTAACTTCTTCGG
LigB12 A1065K rp	CCGAAGAAGTTAAATCCTTTTTGGTTCCATCCGA
LigB12 D1066A fp	GATGGAACCAAAGCGGCTTTAACTTCTTCGGTTAC
LigB12 D1066A rp	GTAACCGAAGAAGTTAAAGCCGCTTTGGTTCCATC
LigB12 E1088A fp	GTGAGTAACGCATCTGCAACGAAAGGATTGGTT
LigB12 E1088A rp	AACCAATCCTTTCGTTGCAGATGCGTTACTCAC

### Protein purification

After being cultivated in LB medium containing 1 mM of IPTG at 20°C overnight, the bacterial cells were disrupted by French press (AIM-AMINCO Spectronic Instruments) at 12,000 psi. The cell lysates were centrifuged at 14,000 rpm for 30 minutes, and the cell-free supernatants were loaded onto Ni^2+^-NTA affinity columns or glutathione agarose to purify the proteins with corresponding affinity tags. For GST-LigB12 and GST-FgαCC8, glutathione agarose pre-equilibrated with PBS buffer (pH = 7.5) was used for purification as previously described [29]. Additionally, His-SUMO tagged FgαCC truncations and Lig proteins were purified by the Ni^2+^-NTA resin, and the His-SUMO tag was further removed by digesting with the SUMO-specific protease Ulp-1 at 4°C overnight followed by application to a second Ni^2+^-NTA column [30]. The tag free proteins were eventually subjected to size exclusion chromatography to obtain higher purity proteins for the following experiments.

### ELISA binding assays

To examine the binding affinity of various FgαCC truncates to Lig proteins, 1 μM of FgαCC1, FgαCC2, FgαCC3, FgαCC4, FgαCC5, FgαCC6, FgαCC7 and FgαCC8 were coated on microtiter wells in 0.1 M NaHCO_3_ (pH 9.4) coating buffer at 4°C overnight. Full length FgαCC and BSA were included in the binding assay as a positive and a negative control. To investigate the critical residues of FgαCC8 mediating the interaction with LigB, different FgαCC8 mutants K606A, R608A, V610A, R611A, I613A, H614A, L618A and K620A were individually immobilized on the microtiter wells using the conditions stated above. All microtiter plates were blocked with PBS buffer containing 3% BSA at 37°C for one hour, and then serial two -fold dilutions of GST tagged LigB12 (0, 0.094, 0.188, 0.375, 0.75, 1.5 and 3 μM) were applied to FgαCC truncate coated wells for an additional one hour at 37°C. To determine the critical FgαCC8-interacting residues on LigB12, 1 μM of LigB12 mutants (F1054A, D1061N, A1065K, D1066A and E1088A) were individually immobilized on microtiter plates. Another mutant P1040C/F1053C [27] and BSA were also included as controls. Subsequently, various concentrations of GST-FgαCC8 (0, 0.094, 0.188, 0.375, 0.75, 1.5 and 3 μM) were added to LigB12 mutant coated wells. To examine the effect of pH on LigB12 binding to FgαCC8, GST tagged LigB12 was prepared in phosphate buffers (150 mM of NaCl) with pH ranging from 5 to 9 and diluted into various concentrations (0, 0.156, 0.313, 0.625, 1.25, 2.5 and 5 μM). For measuring the influence of ionic strength on LigB12-FgαCC8 interaction, different concentrations of GST tagged LigB12 (5 μM ~ 0.156 μM) was prepared in phosphate buffers with salt gradients from 1200 mM, 600 mM, 300 mM, 150 mM to 75 mM NaCl. These pH- and salt-treated preparations of LigB12 were then individually applied to the wells which had been coated with anti-His tag antibody (1:500) and then saturated with His-Sumo tagged FgαCC8. To ensure that the immobilization level of His-Sumo tagged FgαCC8 from each well were equal, the anti-Sumo tag antibody was used for monitoring the amounts of FgαCC8 in control wells. Between each binding step, the plates were washed with PBS buffer containing 0.05% Tween 20 (0.05% PBS-T) for three times to remove non-specific or unbound molecules. For all experiments, HRP-conjugated rabbit anti-GST IgG antibodies (1:2000) were added to detect the FgαCC -bound LigB12 or LigB-bound FgαCC8. Following more washes with 0.05% PBS-T, 100 μl of 0.2 mg/ml TMB substrate was added to the reaction as a chromogen. Finally, the microtiter plates were read at 630 nm by an ELISA plate reader (Biotek EL-312). The equilibrium dissociation constant (K_D_) was calculated by fitting the data to a dose-response curve using the following binding equation:

Response=ResponseMAX+(ResponseMIN-ResponseMAX)/(1+(x/EC50)^Hillslope)

The EC_50_ for ELISA binding assays was equivalent to the K_D_ for the specific assayed binding interactions.

### Binding kinetics by surface plasmon resonance (SPR)

To characterize the real-time binding events between LigB12 and FgαCC truncations, SPR was performed using a Biacore 3000 instrument (GE Healthcare). In brief, 50 μg/mL tag free LigB12 in 10 mM acetate buffer (pH 4.0) was immobilized on a flow cell of a CM5 sensor chip until reaching a level of 1000 resonance units. The control flow cell was also activated and blocked by the same reagents (NHS-EDC and ethanolamine) used for the LigB12-coated cell except that no Lig protein was added. Serial concentrations of FgαCC truncates (0, 0.047, 0.094, 0.188, 0.375, 0.75, 1.5 and 3 μM of FgαCC3, FgαCC5 and FgαCC8) in PBS buffer were individually injected into the flow cells at a flow rate of 30 μL/min. The chip surface was regenerated by removal of analyte with 10 mM glycine-HCl (pH 3.0). All sensograms were recorded at 25°C and normalized by subtracting the data from the control flow cell. To determine the kinetic parameters (k_on_ and k_off_) and the binding affinity (K_D_) of LigB12-FgαCC interactions, the sensograms were fitted by BIAevaluation 3.1 software using one-step biomolecular association reaction model (1:1 Langmuir model), which gave the optimal mathematical fits with the lowest χ values.

### Interaction of FXIII with LigB12

To examine whether LigB12 could directly bind to FXIII, different concentrations of GST-tagged LigB12 (0, 0.094, 0.188, 0.375, 0.75, 1.5 and 3 μM) were added to FXIII-coated microtiter plates. All FXIII-coated wells were blocked with PBS buffer containing 3% BSA at 37°C for one hour. GST-tagged LigB4 at various concentrations were also applied to FXIII-coated wells as a negative control. After three washes with 0.05% PBS-T, bound LigB proteins were detected by anti-GST antibodies conjugated with HRP as mentioned above.

### Inhibition of FXIII-Fg interaction by LigB12

To test whether LigB could affect the FXIII-Fg interaction, serial dilutions (0, 0.63, 1.25, 2.5, 5, and 10 μM) of LigB12 or LigB4 (negative control) were added to FXIII-coated wells for 1 h at 37°C. Lig proteins lacking affinity tags were prepared from His-SUMO tagged constructs. The His-SUMO tag was removed by digestion and column purification as described in the section on protein purification. His-SUMO tag removal was verified by western blot with an anti-His antibody. The unbound LigB proteins were then removed by three times washes with 0.05% PBS-T. His-Sumo tagged FgαCC (1 μM final) was added to each well for 1 h at 37°C, and bound FgαCC was detected by anti-His antibodies conjugated with HRP. To calculate the relative binding of FgαCC to FXIII in the presence of LigB12, the binding level was normalized in relation to the binding of FgαCC to FXIII in the absence LigB. Binding inhibition was calculated with the following equation:

%Inhibition=InhibitedResponseMAX+(100-InhibitedResponseMAX)/(1+(x/IC50)^Hillslope)

### Inhibition of FXIII-mediated Fg crosslinking by LigB12

To examine if LigB proteins could interfere with FXIII-facilitated cross-linking of Fg, different concentrations of untagged LigB12 (15, 7.5, 3.75 μM) or LigB4 (15 μM) were pre-treated with 0.5 mg/ml of Fg and 0.025 mg/ml of FXIII for 10 min at room temperature. The Fg-FXIII mixture without Lig proteins or with EDTA was also included as a positive and a negative control. Subsequently, 1U of thrombin was added to the mixtures for an additional 30 min at 37°C. All reactions were incubated in 50 mM Tris buffer (pH = 7.4) with 100 mM NaCl and 5 mM CaCl_2_. Finally, the reaction was stopped by boiling for 10 min in SDS-PAGE sample buffer containing 1% b-mercaptoethanol and 4 M urea. The samples were then subjected to 10% SDS-PAGE analysis as previously described [14,31].

### Statistical analysis

GraphPad Prism 6.0 (GraphPad Software, Inc.), ANOVA tests, and *t* tests were used to analyze the data.

## Results

### The very C-terminal 23 amino acid residues of Fg α chain is the binding site for LigB12

**Fig 3:**
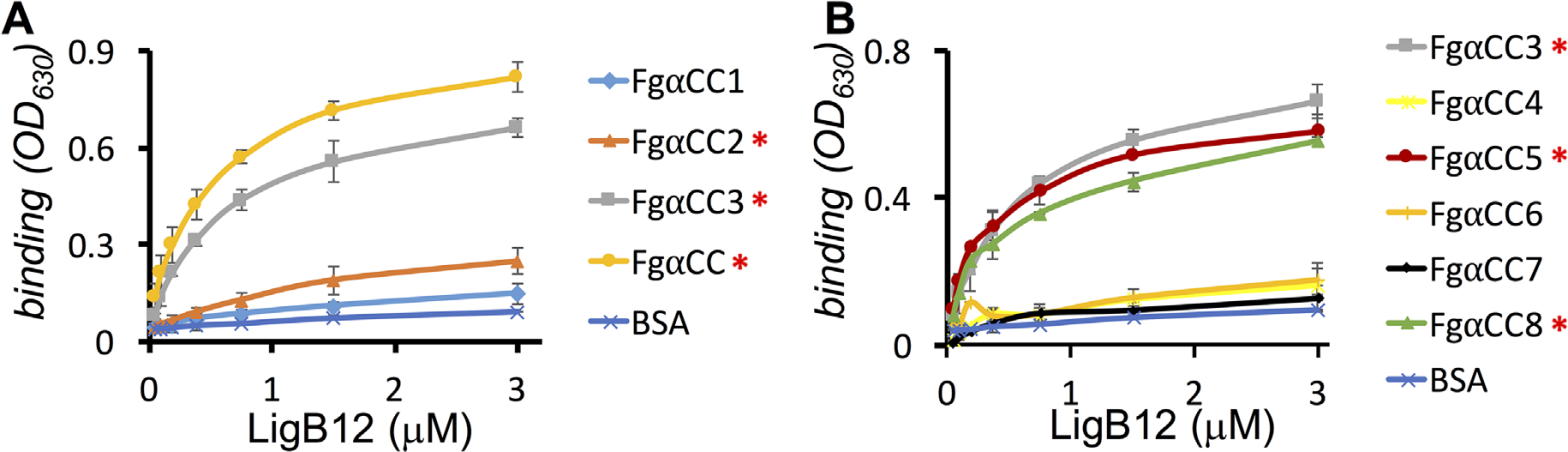
Mapping the binding sites of LigB12 on FgαCC. A series of FgαCC truncates **(A)** FgαCC1, FgαCC2, FgαCC3 and FgαCC **(B)** FgαCC3, FgαCC4, FgαCC5, FgαCC6, FgαCC7 and FgαCC8 (1 αM/well) or BSA (negative control) were immobilized on microtiter plates. Then, various concentrations (0, 0.094, 0.188, 0.375, 0.75, 1.5 and 3 αM) of GST tagged LigB12 were individually applied to FgαCC-coated or BSA-coated wells. The binding of LigB12 to different FgαCC fragments was detected by ELISA using HRP-conjugated anti-GST antibodies. All experiments were conducted in three independent experiments and the results illustrated as the mean +/- 1 standard deviation. Positive binding regions were identified by comparing with BSA and statistically significant (ANOVA test, p < 0.05) differences are marked by an asterisk.

Previously, we identified that LigB12 binds to the C-terminus of Fg α chain (FgαCC) [17]. To further pinpoint the binding site for LigB12, FgαCC was truncated into three fragments based on previously reported structure [32,33]: FgαCC1, FgαCC2 and FgαCC3 as shown in Fig 2. GST-tagged LigB12 was added to microtiter wells coated with different FgαCC truncates including the full-length FgαCC (positive control) and BSA (negative control). As expected, LigB12 bound to full-length FgαCC with the highest affinity (K_D_ = 0.37± 0.05 μM), but not to BSA (Fig 3A). Among all FgαCC truncates, FgαCC3 was most strongly recognized by LigB12 with a calculated K_D_ equal to 0.51± 0.07 μM. FgαCC2 interacted with LigB12 to a much lesser extent, but this interaction was still 2.5-fold greater than the negative control (p<0.05, 3μM of LigB12). In contrast, FgαCC1 showed no significant binding to LigB12 compared to the negative control (p>0.1).

Given the binding of FgαCC3 to LigB12 was stronger than FgαCC2 and this binding was saturated, FgαCC3 was further divided into two fragments, FgαCC4 and FgαCC5 (Fig 2), based on secondary structure prediction server Jpred4 [34]. As indicated in Fig 3B, FgαCC5 bound to LigB12 with great affinity (K_D_ = 0.58± 0.04 μM), while FgαCC4 exhibited no significant binding ability to LigB12 compared to the negative control (p>0.1, 3μM of LigB12). Both tPA and PLG bind to a region covered by FgαCC4 [35]. Interestingly, Lin et al. [17] found that LigB12 does not compete with the binding of tPA or PLG to Fg. In agreement with those findings, we demonstrated that LigB12 preferentially binds to FgαCC5, which is not the binding site for tPA and PLG.

To fine map the minimal binding site, FgαCC5 was eventually truncated into three small fragments, FgαCC6, FgαCC7 and FgαCC8 (Fig 2). As opposed to the binding of FgαCC3 or FgαCC5 to LigB, saturation binding was almost reached for the FgαCC8-LigB12 interaction with K_D_ equal to 0.76± 0.06 μM. The interaction between FgαCC8 and LigB12 was weaker than the interaction between FgαCC3 and LigB12 (p<0.05). On the other hand, neither FgαCC6 nor FgαCC7 could be recognized by LigB12, the interactions of which was not different from the negative control (p>0.1, 3μM of LigB12). FgαCC6 encompassed the RGD motif for the association with platelet integrin α_IIb_β3. Consistent with our previous work [17], LigB12 did not directly bind to the RGD motif within FgαCC6. The findings demonstrate that the very C-terminal 23 amino acid residues of Fg α chain (FgαCC8) is the smallest Fg-derived peptide able to make a significant contribution to the binding site for LigB12.

### LigB12 binds to FgαCC truncations with submicromolar affinities

**Fig 4:**
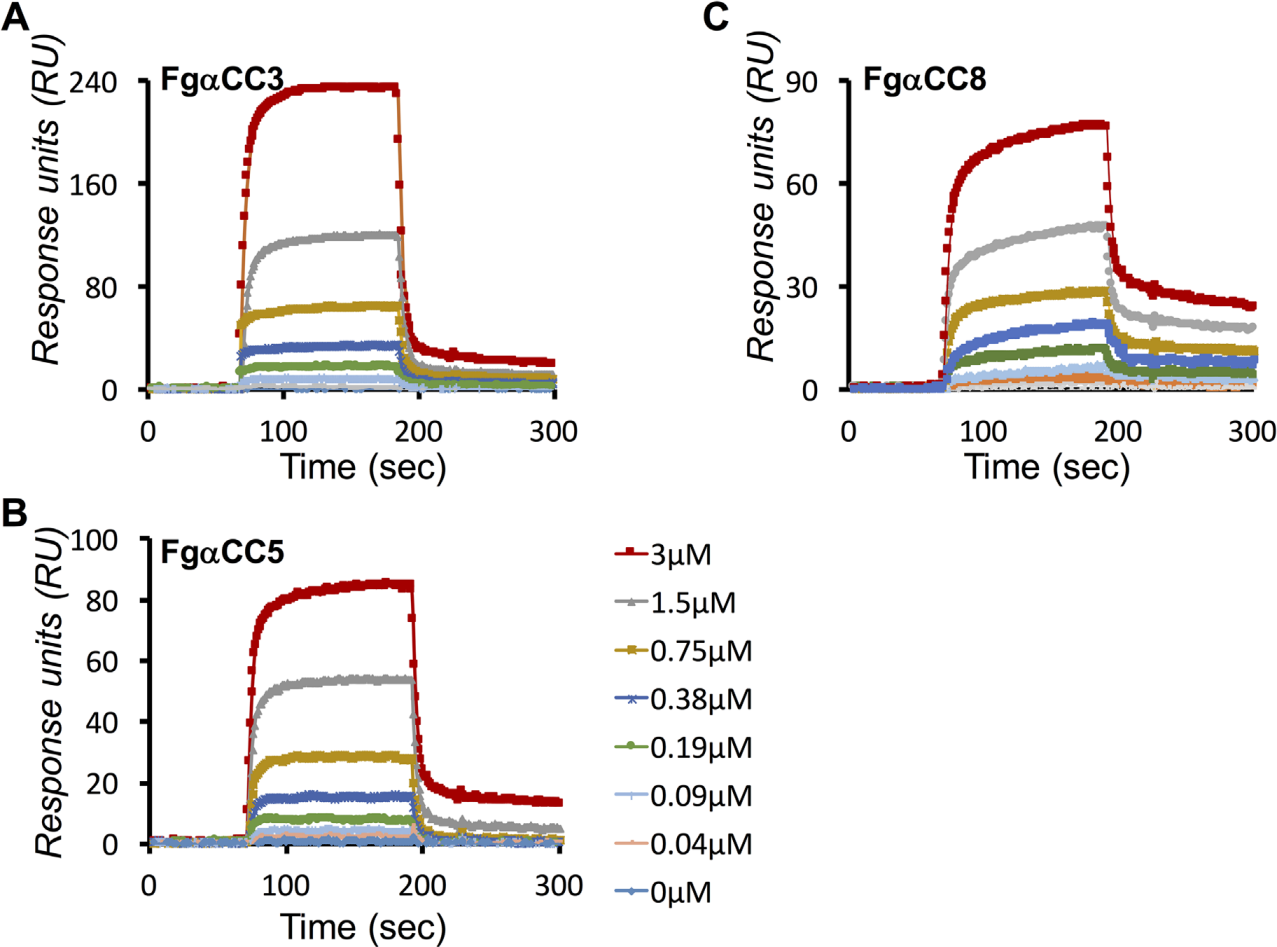
Characterization of binding kinetics of LigB12-FgαCC interactions. SPR analysis of LigB12-FgαCC interactions was conducted by flowing **(A)** FgαCC3 **(B)** FgαCC5 and **(C)** FgαCC8 at 0, 0.047, 0.094, 0.188, 0.375, 0.75, 1.5 and 3 μM through LigB12-immobilized CM5 sensor chip. The measured k_on_, k_off_, and K_D_ values for LigB12-FgαCC3 interaction are 4.81 × 10^4^ ± 0.15 M^-1^ s^-1^, 3.39 × 10^−2^ ± 0.22 s^-1^, 0.704 ± 0.14 μM, respectively. The kinetic parameters of LigB12-FgαCC5 interaction were measured as 3.69 × 10^4^ ± 0.67 M^-1^ s^-1^, 3.84 × 10^−2^ ± 0.14 s^-1^, 1.040 ± 0.21 μM. For LigB12-FgαCC8 interaction, the parameters of binding kinetics were measured as k_on_, 3.72 × 10^4^ ± 0.16 M^-1^ s^-1^, k_off_, 3.57 × 10^−2^ ± 0.08 s^-1^, K_D_, 0.959 ± 0.05 μM.

To accurately characterize the real-time binding kinetics of LigB-FgαCC interactions, the binding of LigB12 to FgαCC truncations (FgαCC3, FgαCC5 and FgαCC8) was analyzed by surface plasmon resonance (SPR). Each FgαCC fragment at different concentrations was passed through a LigB12-coated CM5 sensor chip. The association and dissociation curves were then obtained to calculate association (k_on_) and dissociation rate constants (k_off_) by fitting the sensograms with the 1:1 Langmuir binding model. As shown in Fig 4A and Table 2, FgαCC3 presented a fast association followed by a fast dissociation with LigB12. The binding affinity of the FgαCC3-LigB12 interaction (k_on_ = 4.81 × 10^4^ ± 0.15 M^-1^ s^-1^, k_off_ = 3.39 × 10^−2^ ± 0.22 s^-1^, K_D_ = 0.704 ± 0.14 μM) was 1.8-fold weaker than the interaction between full-length FgαCC and LigB12 (Table 2). The decrease in affinity might be attributed to the loss of the minor LigB12 binding site (FgαCC2) on FgαCC3. In addition, FgαCC5 exhibited a fast-on yet slow-off binding pattern to LigB12 with kinetic parameters k_on_ = 3.69 × 10^4^ ± 0.67 M^-1^ s^-1^, k_off_ = 3.84 × 10^−2^ ± 0.14 s^-1^, K_D_ = 1.04 ± 0.21 μM (Fig 4B). On the other hand, the smallest LigB12-binding construct, FgαCC8, displayed an even slower dissociation to LigB12 with k_on_ = 3.72 × 10^4^ ± 0.16 M^-1^ s^-1^, k_off_ = 3.57 × 10^−2^ ± 0.08 s^-1^, K_D_ = 0.959 ± 0.05 μM (Fig 4C). Although the binding affinity of FgαCC8 to LigB12 was lower than full-length FgαCC, the affinity was still within the sub-micromolar range. Overall, we showed that the minimal binding site (FgαCC8) on Fg α chain maintained a great affinity to LigB12.

**Table 2:** Kinetic parameters of LigB12-Fg interactions.

	SPR			ELISA
	K_on_ (M^-1^ s^-1^)	K_off_ (s^-1^)	K_D_ (μM)	K_D_ (μM)
FgαCC^#^	5.03 × 10^4^ ± 0.03	1.88 × 10^−2^ ± 0.26	0.385 ± 0.09	0.37 ± 0.05
FgαCC3	4.81 × 10^4^ ± 0.15	3.39 × 10^−2^ ± 0.22	0.704 ± 0.14*	0.51 ± 0.07*
FgαCC5	3.69 × 10^4^ ± 0.67	3.84 × 10^−2^ ± 0.14	1.040 ± 0.21	0.58 ± 0.04
FgαCC8	3.72 × 10^4^ ± 0.16	3.57 × 10^−2^ ± 0.08	0.959 ± 0.05 **	0.76 ± 0.06 **

All values represent the mean ± 1 standard deviation of three independent experiments.

^#^The kinetic data of FgαCC-LigB12 interaction is cited from Lin et al., 2011 [17].

*The dissociation constants (K_D_) of FgαCC3-LigB12 interaction derived from SPR and ELISA are both significantly higher than K_D_ of FgαCC-LigB12 interaction (*t* test, p< 0.05).

**The dissociation constants (K_D_) of FgαCC8-LigB12 interaction obtained from SPR and ELISA are both significantly higher than K_D_ of FgαCC-LigB12 interaction (*t* test, p< 0.05).

### The LigB12-FgαCC8 interaction is mediated by electrostatic and hydrophobic forces

**Fig 5:**
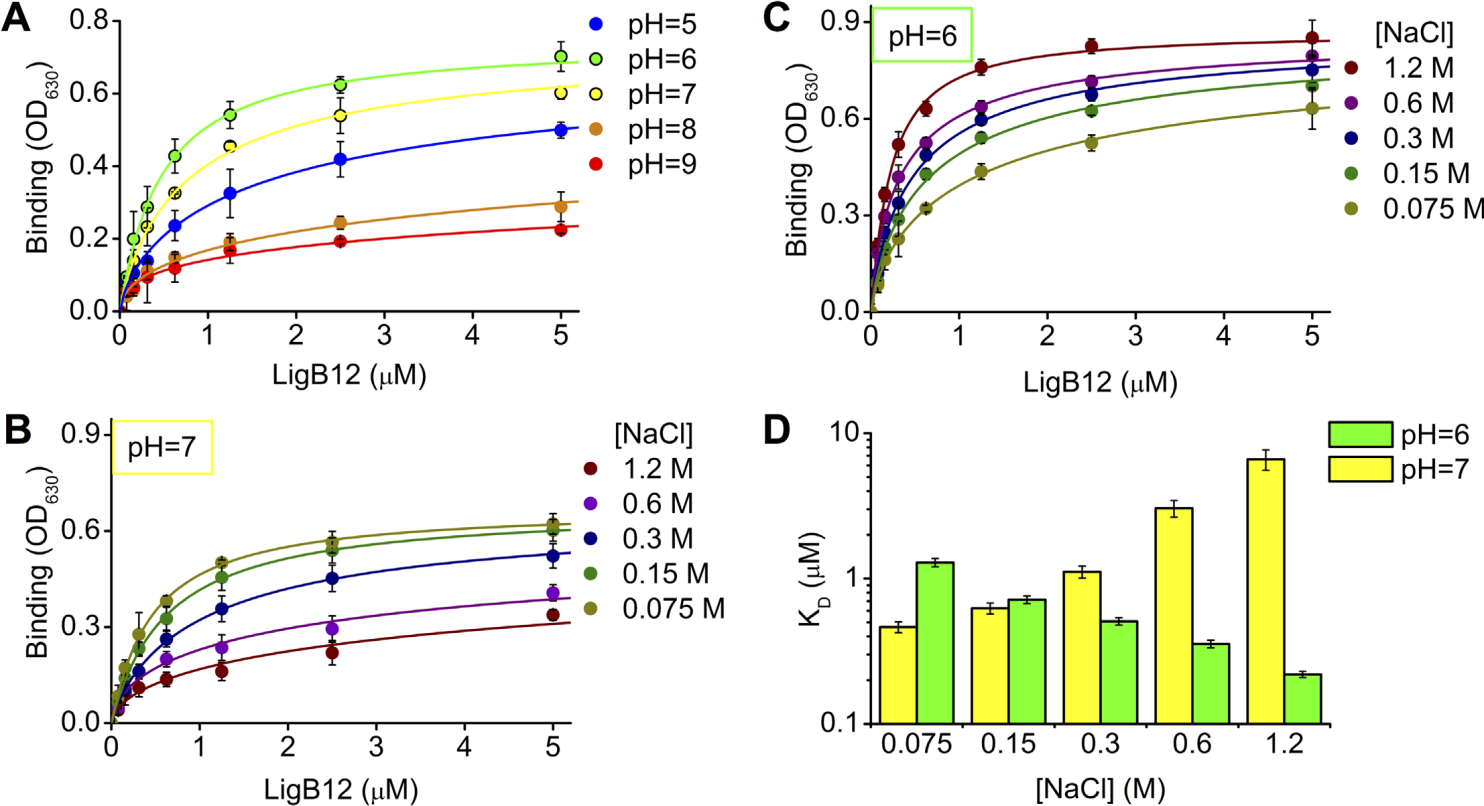
Effect of different pH conditions and salt concentrations on the binding of LigB12 to FgαCC8. For all experiments, anti-His monoclonal antibody was immobilized on each well to provide a specific binding site for His-Sumo tagged FgαCC8 and to allow FgαCC8 to be fully accessible to LigB12. **(A)** Various concentrations of GST tagged LigB12 (0, 0.156, 0.313, 0.625, 1.25, 2.5 and 5 μM) in 0.1 M of potassium phosphate buffer at different pH values (5, 6, 7, 8 and 9) were applied to FgαCC8-immobilized wells (1 μg/well). (**B)** and **(C)** Various concentrations of GST tagged LigB12 (0, 0.156, 0.313, 0.625, 1.25, 2.5 and 5 μM) in 0.1 M of potassium phosphate buffer at (**B)** pH = 7 or (**C)** pH = 6 were prepared under different salt conditions (0.075 M, 0.15 M, 0.3 M, 0.6 M and 1.2 M of NaCl), and then added to FgαCC8-immobilized wells. The binding affinity of GST-LigB12 to FgαCC8 was subsequently detected by ELISA using HRP-conjugated anti-GST antibodies. (**D)** The binding affinity of LigB12 to FgαCC8 (K_D_) at pH 7 (in yellow) and pH 6 (in green) were graphed for each NaCl concentration. The dissociation constants (K_D_) were calculated by fitting the binding curves from (**B)** and **(C)** with the equation described in materials and methods. The mean +/- 1 standard deviation shown in the graph was derived from three independent experiments.

The theoretical pI of 12.02 for FgαCC8 (calculated using the Protein Calculator version 3.4 (Putnam, C.D., 2013, http://protcalc.sourceforge.net/) is high due to the four positively charged side chains and absence of negatively charged side chains. The theoretical net charge of FgαCC8 and LigB12 at pH 5, 6, 7, 8, and 9 were calculated and plotted in S1 Fig. The largest charge difference (>4) between the two proteins was found at pH 6. To determine if the LigB12-FgαCC8 interaction is mediated by charge-charge interactions, we tested the binding of LigB12 to FgαCC8 in phosphate buffers with different pH values by ELISA (150 mM NaCl). Interestingly, the binding affinity of LigB12 to FgαCC8 reached the maximum (K_D_ = 0.47 μM) at pH 6 (Fig 5A). At pH 7 or pH 5, the LigB12-FgαCC8 interaction was weaker (K_D_ = 0.82 μM at pH 7; K_D_ = 1.83 μM at pH 5) but still indicated prominent binding. An increase in the pH to 8 or 9 resulted a drop in the binding of LigB12 to FgαCC8 to near basal level (similar to the binding of LigB12 to BSA at pH 7, Fig 3). The ELISA LigB12-FgαCC8 interaction response is highly pH-dependent suggesting that electrostatic forces contribute to the LigB12-FgαCC8 interaction. To determine whether the ionic strength also had an effect on the interaction, we further assayed the binding of LigB12 to FgαCC8 in buffers containing various salt concentrations. At pH 7 (Fig 5B), the greatest binding affinity of LigB12 to FgαCC8 appeared when NaCl concentration was 75 mM (K_D_ = 0.46 μM). In addition, at the 150mM NaCl concentration, LigB12-FgαCC8 interaction was slightly weaker (K_D_ = 0.62 μM) than the affinity in the buffer containing 75mM NaCl. As the salt concentration further increased, the binding affinity gradually dropped and reached its weakest point in the buffer containing 1200 mM NaCl (K_D_ = 6.61 μM) (Fig 5D). Unexpectedly, at pH 6 (Fig 5C), the binding affinity was greater in high salt conditions (K_D_ = 0.22 μM, 1200 mM NaCl). Then, the interaction gradually weakened with decreasing salt concentrations. The affinity reached the minimum (K_D_ = 1.29 μM) when the NaCl concentration was 75 mM (Fig 5D). To be noted, the structure of LigB12 was not affected in the buffers at different pH or various salt conditions (S2A and S2B Fig). All these findings suggested that not only electrostatic forces but also hydrophobic interactions contribute to the binding of LigB12 to FgαCC8.

### Charged residues and nonpolar residues are critical for LigB12-FgαCC8 interaction

**Fig 6:**
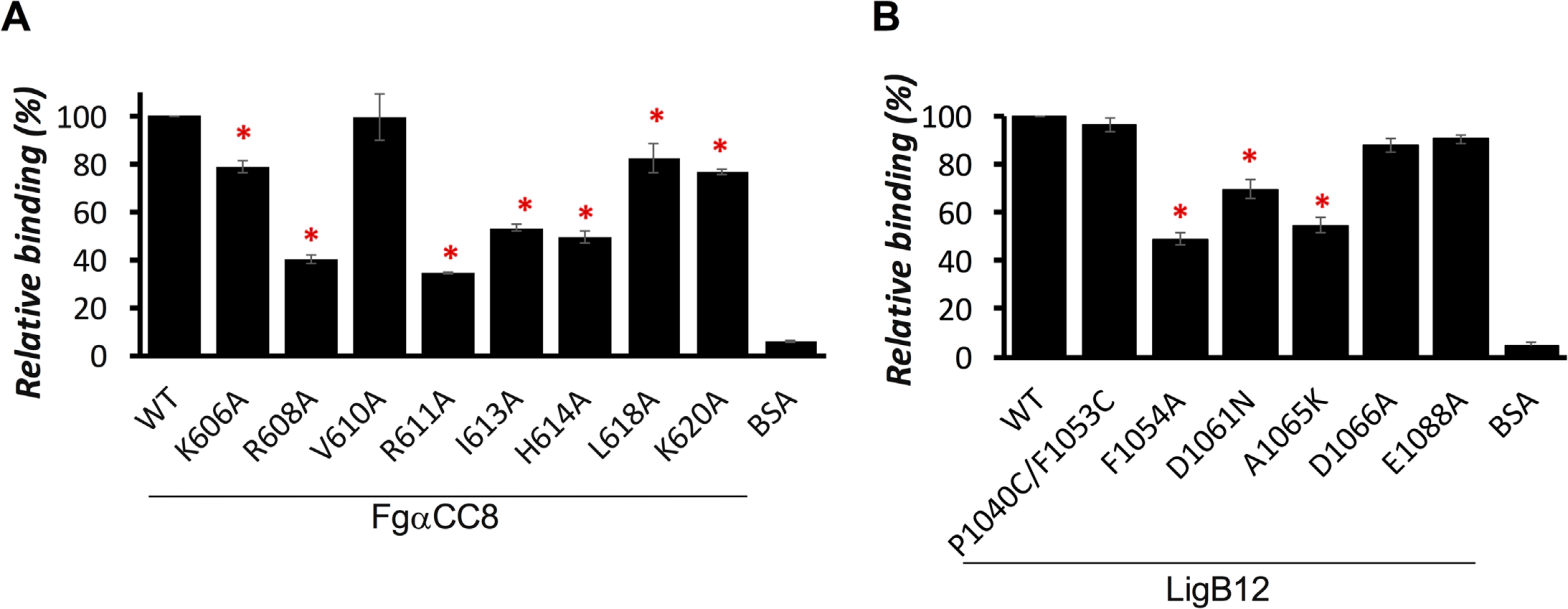
Identification of key amino acids contributing to the LigB12-FgαCC8 interaction. **(A)** The binding affinities of FgαCC8 wild-type (WT) and eight FgαCC8 mutants to LigB12 were measured by ELISA. 5 μM of GST tagged LigB12 were added to WT or FgαCC8 mutants or BSA (negative control) coated wells (1 μM/well). The relative binding (%) of LigB12 to each FgαCC8 mutant was calculated in relation to FgαCC8 WT. (**B)** The binding affinities of LigB12 wild-type (WT) and six LigB12 mutants to FgαCC8 were measured by ELISA. 5 μM of GST tagged FgαCC8 were added to WT or LigB12 mutants or BSA (negative control) coated wells (1 μM/well). The relative binding (%) of FgαCC8 to each LigB12 mutant was calculated in relation to LigB12 WT. The mean +/- 1 standard deviation shown in the graph was derived from three independent experiments. For **(A)** and **(B)**, the mutants that significantly reduced binding as opposed to WT are marked by an asterisk (ANOVA test, p<0.05).

Based on our buffer screening, the key amino acids responsible for the LigB12-FgαCC8 interaction are likely to either be charged or hydrophobic in nature. From FgαCC8, five basic amino acids (K606, R608, R611, H614 and K620) and three aliphatic amino acids (V610, I613 and L618) were targeted in the construction of single alanine mutants. The FgαCC8 mutated fragments maintained a theoretical pI close to 12 (similar to wild-type (WT) FgαCC8) with the exception of R608A and R611A (pI = 11.17) (S3A Fig). In addition, the alanine mutants of the five basic amino acids had an increase in hydropathicity and the alanine mutants of the three aliphatic amino acids had a decrease in hydropathicity from WT FgαCC8 (S3A Fig). All these mutations had similar secondary structures as WT FgαCC8 (S4A Fig). ELISA binding assays were performed with all of the alanine mutants immobilized on microtiter plates to examine the effect of replaced side chain on the LigB12-FgαCC8 interaction. Various concentrations of GST-tagged LigB12 were applied to FgαCC8 mutant-coated wells and the binding was assayed by ELISA. The binding of LigB12 to WT FgαCC8 or BSA was also included as positive and negative controls, respectively. As shown in Fig 6A, R608A and R611A showed dramatic abolishment of LigB12 binding ability (>60% reduction). These two residues were associated with the largest differences in pI and hydropathicity from the WT Fg fragment (S3A Fig). H614A also had 50% reduction of binding to LigB12. On the other hand, the binding of LigB12 to K606A and K620A was only slightly decreased (20–23% reduction) compared to WT. Similar to H614A, I613A showed 47% reduction of binding to LigB12 (Fig 6A). In contrast, L618A only reduced the binding by 12% compared to WT, and V610A did not decrease the binding to LigB12 to any significant degree (p>0.1). In summary, the binding results suggest that R608, R611, I613 and H614 are important for the interaction of FgαCC8 with LigB12.

Considering that three positively charged residues of FgαCC8 were important for the LigB12-FgαCC8 interaction, we postulated that the three negatively charged residues within the main LigB12 domain might be involved in this interaction. The three LigB12 mutants (D1061N, D1066A and E1088A), which increased the theoretical pI to 9.25 from 8.5 for WT LigB12 (S3B Fig), were coated on microtiter wells to test for FgαCC8 binding ability through ELISA. The binding of FgαCC8 to WT LigB12 or BSA was also included as controls. Interestingly, D1061N was the only mutant showing >30% reduction of binding to FgαCC8, while D1066A and E1088A had minor reductions (10~12%) of binding (Fig 6B). Taking advantage of the high resolution structure of LigB12 [27], we were able to identify neighboring residues of D1061 with highly surface accessible side chains that might also contribute to FgαCC8 binding. Two residues located on the surface near D1061, F1054 and A1065, are not present in the other eleven LigB domains that lack the ability to bind FgαCC8. Mutations, F1054A and A1065K, were chosen from residues present at the homologous location in other LigB domains. In addition, the core-facing F1053 was included in the mutagenesis study as a control. Instead of using the poorly-thermostable F1053A mutant, we performed the binding experiment with the P1040C/F1053C mutant which was previously shown to be properly folded and to have thermostability near WT [27,36]. The theoretical pI and hydropathicity for all of the LigB12 mutants is plotted in S3B Fig. In addition, all LigB12 mutations had similar secondary structures as WT LigB12 (S4B Fig). As expected, the binding of P1040C/F1053C to FgαCC8 was not significantly different from WT (p>0.1) (Fig 6B). Both F1054A and A1065K showed a decrease in binding to FgαCC8 (51% and 46% reduction). In conclusion, our ELISA analysis using the surface mutants of LigB12 suggests that D1061, F1054 and A1065 play a major role in the interaction of LigB12 with FgαCC8.

### LigB12 interferes with FXIII binding to Fg and FXIII-mediated cross-linking of Fg

**Fig 7:**
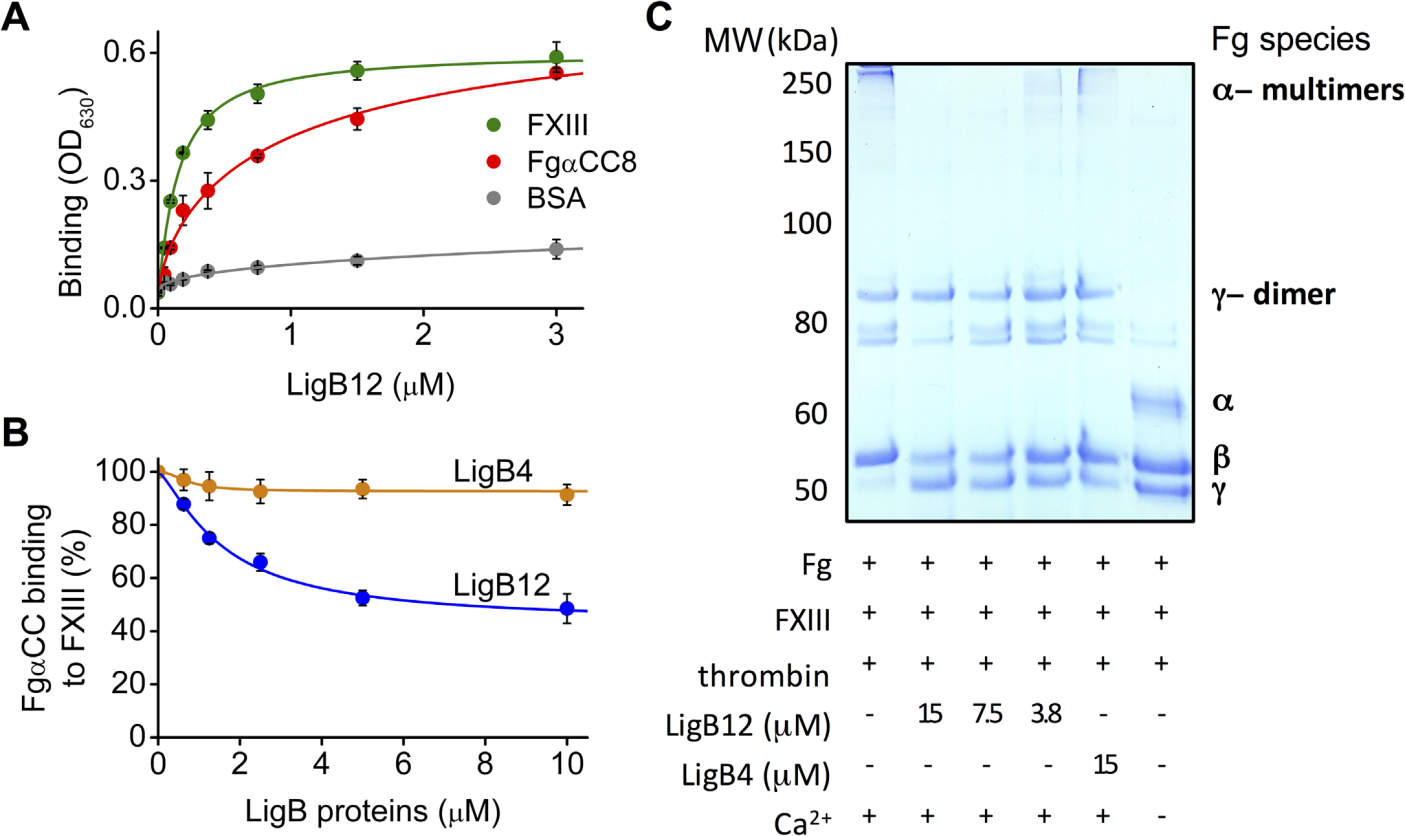
Binding of LigB12 to FXIII/Fg interferes with FXIII-mediated Fg cross-linking. **(A)** Various concentrations of GST tagged LigB12 (0, 0.094, 0.188, 0.375, 0.75, 1.5 and 3 μM) were added to FXIII (green), FgαCC8 (positive control; red) and BSA (negative control; gray) immobilized wells. The binding of LigB12 to FXIII was measured by ELISA. (**B)** Different concentrations (0, 0.63, 1.25, 2.5, 5, and 10 μM) of LigB12 (blue) or LigB4 (orange) were added to FXIII-coated wells. After the unbound LigB proteins were removed, His-SUMO-tagged FgαCC (1μM) was added to the well for a 1 hr incubation. The binding of FgαCC to FXIII was then detected by anti-His antibodies conjugated with HRP. The relative binding affinity (%) of FgαCC to FXIII in the presence of Lig proteins was calculated in relation to the binding of FgαCC to FXIII in the absence LigB proteins. For **(A)** and **(B)**, experiments were conducted in three independent trails and the mean +/- 1 standard deviation is shown in the graph. (**C)** The effect of LigB proteins on FXIII-assisted Fg cross-linking. Various concentrations of LigB12 (15, 7.5, 3.75 μM) or LigB4 (15 μM) were pre-treated with 0.5 mg/ml of soluble Fg and 0.025 mg/ml of FXIII for 10 min prior to the addition of 1U of thrombin. The reaction was conducted at 37°C for 30 min in 50 mM Tris buffer (pH = 7.4) containing 100 mM NaCl and 5 mM CaCl_2_. Mixtures without LigB protein (positive control) or without CaCl_2_ (negative control) were also included. The SDS-PAGE analysis shown here is a representative of three independent experiments.

LigB12 inhibits blood clot formation by interfering with the lateral aggregation of fibrin [17]. At this stage of fibrin clot formation, the αC domain of Fg tends to associate with another αC domain on a neighboring Fg, which provides a proper conformation for subsequent cross-linking catalyzed by FXIII [37]. FXIII is converted to its active transglutaminase form, FXIIIa, by thrombin (in the presence of Ca^2+^). FXIIIa catalyzes the formation of covalent peptide bonds between Lys and Gln side chains from Fg. Eventually, the cross-linked Fg α and γ chains form an insoluble fibrin network to stop the hemorrhage [38]. In addition, Smith et al. [39] showed that FXIII could bind to FgαCC with submicromolar affinity. Based on these previous results, we hypothesized that LigB12 might be able to impair the FXIII-Fg interaction through a direct interaction with FXIII. To this end, the direct ELISA binding assay was performed by applying LigB12 to FXIII-coated microtiter wells. LigB12 was also added to FgαCC8- or BSA-coated wells as a control. As expected, LigB12 was strongly bound by FgαCC8 but not BSA (Fig 7A). FXIII exhibited an even stronger binding affinity to LigB12 (K_D_ = 0.15 ± 0.015 μM).

To investigate whether the tight binding of LigB12 to FXIII could interfere with the FXIII-FgαCC interaction, different concentrations of LigB12 were applied to FXIII-coated wells. LigB4, which does not bind to either FXIII or FgαCC (S5 Fig), was included as a negative control. After the unbound LigB proteins were removed, the ability of His-SUMO FgαCC binding to FXIII was examined. According to the LigB4 dose inhibition curve shown in Fig 7B, increasing concentrations of LigB4 could inhibit FgαCC binding to FXIII by at most 7.3 ± 2.9% suggesting that the presence of LigB4 had almost no effect on the binding of FgαCC to FXIII. In contrast, increasing concentrations of LigB12 showed a more significant inhibition of FgαCC binding to FXIII. The LigB12 dose inhibition curve fit to a 56.7 ± 8.2% reduction in the binding of FgαCC to FXIII at high LigB12 concentrations. Because FgαCC binding sites are present on the immobilized FXIII and also potentially exposed on the FXIII-bound LigB12, the inhibition assay does not rule out the possibility that some FgαCC might bind directly to LigB12 thereby reducing the apparent blocking effects of LigB12 on the FXIII-FgαCC interaction.

Finally, we examined whether Lig proteins could affect FXIII-mediated cross-linking of Fg. LigB12 or LigB4 (negative control) was pre-incubated with FXIII and soluble Fg before being mixed with thrombin to initiate the cross-linking. The reaction was conducted at 37°C for 30 min in CaCl_2_ containing buffer and then boiled for SDS-PAGE analysis. The reaction mixtures without LigB proteins (positive control) or without CaCl_2_ (negative control) were also included. In the positive control, both α and γ chains were fully cross-linked and migrated as high molecular weight α- multimers and γ-dimers, while the inert β chain stayed as a monomer (Fig 7C). In the negative control, the cross-linking reaction was completely blocked by EDTA. As a result, all α and γ chains migrated in their monomeric forms. Notably, LigB12 could partially inhibit polymerization of α chains but could not significantly decrease the dimerization of γ chains. On the other hand, LigB4 did not reduce either α- multimer or γ-dimer formation. To sum up, we have found that LigB12 inhibits fibrin clot formation by interfering with the cross-linking of Fg α chains.

## Discussion

The animal coagulation system is one of the major defense systems to help cope with microbial infections. This primitive system is highly conserved from invertebrates to humans; for example, a clottable protein functioning like Fg is preserved in horseshoe crabs [40]. Once pathogen induced damage to the vasculature occurs, clotting factors are rapidly activated, eventually resulting in fibrin clot formation to wall off the invading microbes. Among all clotting factors, Fg is the primary building unit of the fibrin clot and plays the central role of recruiting thrombin, FXIII and platelets to initiate the clotting cascade. Fg also triggers the activation of the fibrinolytic system by bringing tPA and PLG together. The check and balance of hemostasis and fibrinolysis needs to be well orchestrated; otherwise, the resulting hemorrhage or thrombosis may lead to detrimental consequences [12]. Notably, Fg is primary synthesized by the liver, but it can also be secreted by alveolar type I pneumocytes during lung infections [41]. A hemorrhagic lung provides a perfect niche for *leptospira* to thrive in. Although there was no direct evidence showing the up-regulation of Fg synthesis from *leptospira*-associated pneumonia, microscopic lesions from *leptospira* infected patients did reveal multifocal fibrin deposition and severe hemorrhage [11,42,43]

Bacterial pathogens have evolved a variety of adhesins to interact with hemostatic factors including Fg. Bacterial adhesins, depending on their mechanism of bacterial surface attachment, can be categorized into two groups: Microbial Surface Components Recognizing Adhesive Matrix Molecules (MSCRAMMs) or Secretable Expanded Repertoire Adhesive Molecules (SERAMs) [11,43]. Fg-interacting proteins can target distinct sites on different chains of Fg, but they all antagonize Fg function leading to blockage of the normal coagulation cascade. ClfA and fibronectin binding protein A (FnBPA) both bind to the γC domain to block platelet aggregation by occupying the integrin recognition site. Both proteins also inhibit fibrin polymerization through interference with B knob- b hole interaction [13,44]. SdrG interacts with the N-terminal β chain, the thrombin-targeting site, to inhibit the cleavage of FpB and thus abolish fibrin clot formation [15,45]. The αC domain is a common target for clumping factor B (ClfB) and bone sialoprotein binding protein (Bbp). ClfB binds to the N-terminal flexible region of the αC domain (FgαCN), while Bbp interacts with the C-terminal globular region of the αC domain (FgαCC) [46,47]. One example of a SERAM is the extracellular fibrinogen-binding protein (Efb), which associates with the Fg α chain to reduce leukocyte adherence, thereby disrupting host immune responses [48]. Recently, many leptospiral Fg-binding proteins have been identified [16,18,19], but the specific minimal binding sites on Fg for these adhesins have not yet been elucidated. The leptospiral MSCRAMM, LigB, contains an Ig-like domain structure that is common in many Fg-binding adhesins and has a high affinity for FgαCC [27]. In this study, we further identified that the very C-terminal 23 residues of FgαCC (FgαCC8) can be recognized by LigB12 (Fig 3). Based on the solution structure of FgαCC determined by NMR, FgαCC8 should be a fairly flexible region protruding from a well-folded αC domain [32,33]. The inherent flexibility of FgαCC8 may lead to structural differences in the context of FgαCC and may also be responsible for the weaker binding affinity of FgαCC8 for LigB12. Interestingly, dynamic, unstructured features of Fg are the most common ligands for bacterial adhesins [13,45,46]. We also showed that the LigB12 binding site on FgαCC did not overlap with tPA, PLG or platelet integrin targeting site (Fig 3), which is consistent with our previous findings [17]. Previously, a study by another group identified that Fg binding site on the 9^th^ through 11^th^ Ig-like domains of LigB [20]. While results from our group agree that the 1^st^ through 7^th^ Ig-like domains of LigB have little to no affinity for Fg, our results disagree in the specific Fg-binding sites within the final four LigB Ig-like domains [17,20–22]. One potential reason for the discrepancy could be that the LigB gene used in this study was cloned using serovar Pomona chromosomal DNA while the other study used a LigB gene cloned from serovar Copenhageni chromosomal DNA. The amino acid sequences differ slightly between these two serovars, which could affect the interaction with Fg.

The Fg concentration in plasma ranges between 5.8 to 11.6 μM under normal physiological conditions [49]. To tightly associate with Fg, most bacterial adhesins bind to Fg with sub-micromolar affinity [17,20,45]. Using ELISA and SPR, we demonstrated that the K_D_ of FgαCC8 binding to LigB12 was ~0.76–0.96 μM (Figs 3B and 4C). This affinity is comparable to other adhesin-Fg interactions and implies that the LigB12-FgαCC is physiologically relevant during infection [11,15,44,47,48]. Furthermore, the LigB12-FgαCC interaction is mediated by both electrostatic and hydrophobic forces (Fig 5) and is supported by previous isothermal titration calorimetry data showing that LigB12-FgαCC interactions are driven by both enthalpy and entropy [17]. Local inflammation is frequently associated with extracellular acidosis [50]. For example, in *Pseudomonas* induced pneumonia, lactic acidosis developed in hemorrhagic lung [51]. Local acidosis favors an inflammatory response leading to the suppression of the coagulation system [52]. Inflammation-induced lung acidosis might be the reason that LigB12 evolved to bind much stronger to FgαCC8 at low pH (Fig 5A). Three positive residues from FgαCC8 (R608, R611 and H614) took part in the association with LigB12 (Fig 6A). Particularly, the involvement of H614 in the interaction should explain the strong affinity occurring at pH 6. The pKa of the histidine sidechain is approximately 6, which implicates that FgαCC8 could carry relatively more positive charge when pH is below 6. This positively charged histidine sidechain could interact with aromatic residues and also form hydrogen bonds with polar residues [53,54], resulting in the enhancement of binding to LigB in a pathologically low pH environment. Likewise, a negatively charged amino acid D1061 from LigB12 contributed to the binding as well (Fig 6B). Therefore, the LigB12-FgαCC interaction is less likely to adopt the typical “dock, lock and latch” binding mechanism of SdrG-Fg interaction in which the hydrophobic residues play a main role [15,55]. On the other hand, I613 from FgαCC8, F1054 and A1065 from LigB12 are the major residues responsible for hydrophobic interaction. D1061 is thought to participate in the binding to human tropoelastin (HTE), another host binding partner of LigB, suggesting that Fg and HTE might share the same binding region on LigB12 [28]. Future studies will aim to clarify this competitive interaction between host factors and LigB.

FXIII is a transglutaminase circulating in the plasma as fibrin stabilizing factor. Following the lateral aggregation of Fg, the proper orientation of α and γ chains allows FXIII catalyzed cross-linking to occur, which stabilizes the fibrin clot thereby becoming resistant to chemicals and mechanical force [38]. LigB could interfere with clot formation at the later stage [17] by affecting Fg cross-linking. Here, we showed that LigB12 binds to FXIII and thus suppresses the FXIII-FgαCC interaction (Fig 7A and 7B). The interaction of LigB12 with FXIII is a potential mechanism of how LigB can disrupt the FXIII-mediated cross-linking of Fg α chains (Fig 7C). Recently, the FgαCC1 region has been identified to contain the majority of the FXIII and FXIIIa binding sites [39]. The FXIII-binding site is thought to be located on a 55 amino acid stretch of FgαC with 34 amino acids on FgαCC. Here, we show that FgαCC retains a portion of its binding affinity for FXIII despite having a reduced interaction site. The presence of LigB12 partially inhibits the polymerization of Fgα chains suggesting that FXIII function is disrupted by LigB12 and that the Fgα-FXIII interaction might also be disrupted by LigB12. In the current study, the LigB12-binding site on the Fg α chain was mapped to the C-terminus of FgαCC (defined as FgαCC8), a site that is distinct from the FXIII-binding site. Given the higher affinity of LigB12 for FXIII than for Fg, LigB12-dependent block of the FXIII-FgαCC interaction was assessed using the tighter LigB12-FXIII interaction to inhibit FXIII-FgαCC formation. The ability of LigB12 to reduce maximal FgαCC binding to FXIII by more than 50% in this assay suggests that LigB12 and Fg compete with each other for the same binding region on FXIII. One potential working model is that LigB from the surface of *Leptospira* interacts with both FXIII and Fg in order to disrupt the coagulation pathway (Fig 1). After being hijacked by *Leptospira*, FXIII loses some ability to cross-link Fg polymers, which further blocks the fibrin clot formation. LigB targeting to a specific site on the Fg α chain could have a negative impact on FXIII-mediated retention of red blood cells, which destabilize the thrombus and might further facilitate the dissemination of *Leptospira* [56]. Previously, LigB was shown to reduce platelet adhesion and aggregation by interfering with the Fg-integrin interaction [17]. Several groups have also found that *Leptospira* could diminish the fibrin clot by either inhibiting thrombin activity or by activating fibrinolysis system, although this abolishment per se was mediated by different leptospiral adhesins [18,57]. Taken together, multiple steps of the blood coagulation pathway could be modulated by leptospiral surface proteins, which should promote the systemic spreading of spirochetes and potentially lead to fatal pulmonary hemorrhage.

In conclusion, we demonstrated that FgαCC8 was the minimal binding site for LigB12. For the first time, the critical residues contributing to the association of leptospiral adhesins with Fg were revealed. In addition, we showed a potential mechanism of LigB interference with fibrin clot formation. Taken together, this study provides a better understanding of host-pathogen interaction and has the potential to aid the development of future leptospirosis therapies.

## Supporting Information

S1 Fig Effect of pH on LigB12 and FgαCC8 overall charge. The primary sequence-based charge values for LigB12 and FgαCC8 are plotted for the pH conditions used to assess the pH-dependence of the LigB12-FgαCC8 binding interaction in ELISA studies (Fig 5A). The pH-specific charge for the proteins was calculated using the Protein Calculator version 3.4 (Putnam, C.D., 2013, http://protcalc.sourceforge.net/). The difference between the charge of the LigB12 and FgαCC8 at each pH is shown as a column chart relative to the charge difference at pH 8 (+3.1, the smallest difference). The largest LigB12-FgαCC8 charge difference occurs at pH 6.
(TIF)

S2 Fig Secondary structure analysis for LigB12 under different buffer conditions. Far-UV circular dichroism analysis of **(A)** wild-type LigB12 in PBS buffers with different pH and **(B)** wild-type LigB12 in phosphate buffers with various concentrations of NaCl. The molar ellipticity, θ, was measured from 200 nm to 240 nm for 10μM of each protein at room temperature.
(TIF)

S3 Fig Parameters computed for mutant FgαCC8 and LigB12 primary sequences. The grand average of hydropathicity (GRAVY) index and theoretical pI are plotted in blue for **(A)** wild-type FgαCC8 and **(B)** wild-type LigB12. The position of mutants are also annotated on the plot of GRAVY index vs. pI. The GRAVY index was calculated using the GRAVY Calculator (Fuchs, S., 2011, http://www.gravy-calculator.de/) and the theoretical pI was calculated using the Protein Calculator version 3.4 (Putnam, C.D., 2013, http://protcalc.sourceforge.net/).
(TIF)

S4 Fig Secondary structure analysis for LigB12, FgCC8 and their mutants. Far-UV circular dichroism analysis of **(A)** wild-type and mutant FgCC8 in PBS buffer, **(B)** wild-type and mutant LigB12 in PBS buffer. The molar ellipticity, θ, was measured from 200 nm to 240 nm for 10μM of each protein at room temperature.
(TIF)

S5 Fig The binding of LigB proteins to FXIII and FgCC8. GST tagged LigB12 and LigB4 (3 μM) were added to FXIII, FgαCC and BSA (negative control) immobilized wells. The binding of LigB12 or LigB4 to FXIII or FgαCC was measured by ELISA using HRP-conjugated anti-GST antibodies. The mean +/- 1 standard deviation shown in the graph was derived from three independent experiments. The significant binding of LigB12 to FXIII or FgαCC as opposed to BSA control (ANOVA test, p < 0.05) was marked by an asterisk. LigB4 did not show any significant binding to either FXIII or FgαCC (ANOVA test, p > 0.1).
(TIF)
