# Supplementary figures and images for: Leptospira Immunoglobulin-Like Protein B (LigB) Binds to Both the C-Terminal 23 Amino Acids of Fibrinogen αC Domain and Factor XIII: Insight into the Mechanism of LigB-Mediated Blockage of Fibrinogen α Chain Cross-Linking
Source: PLoS Negl Trop Dis. 2016 Sep 13;10(9):e0004974. doi: 10.1371/journal.pntd.0004974 (PMC5021285; doi:10.1371/journal.pntd.0004974)

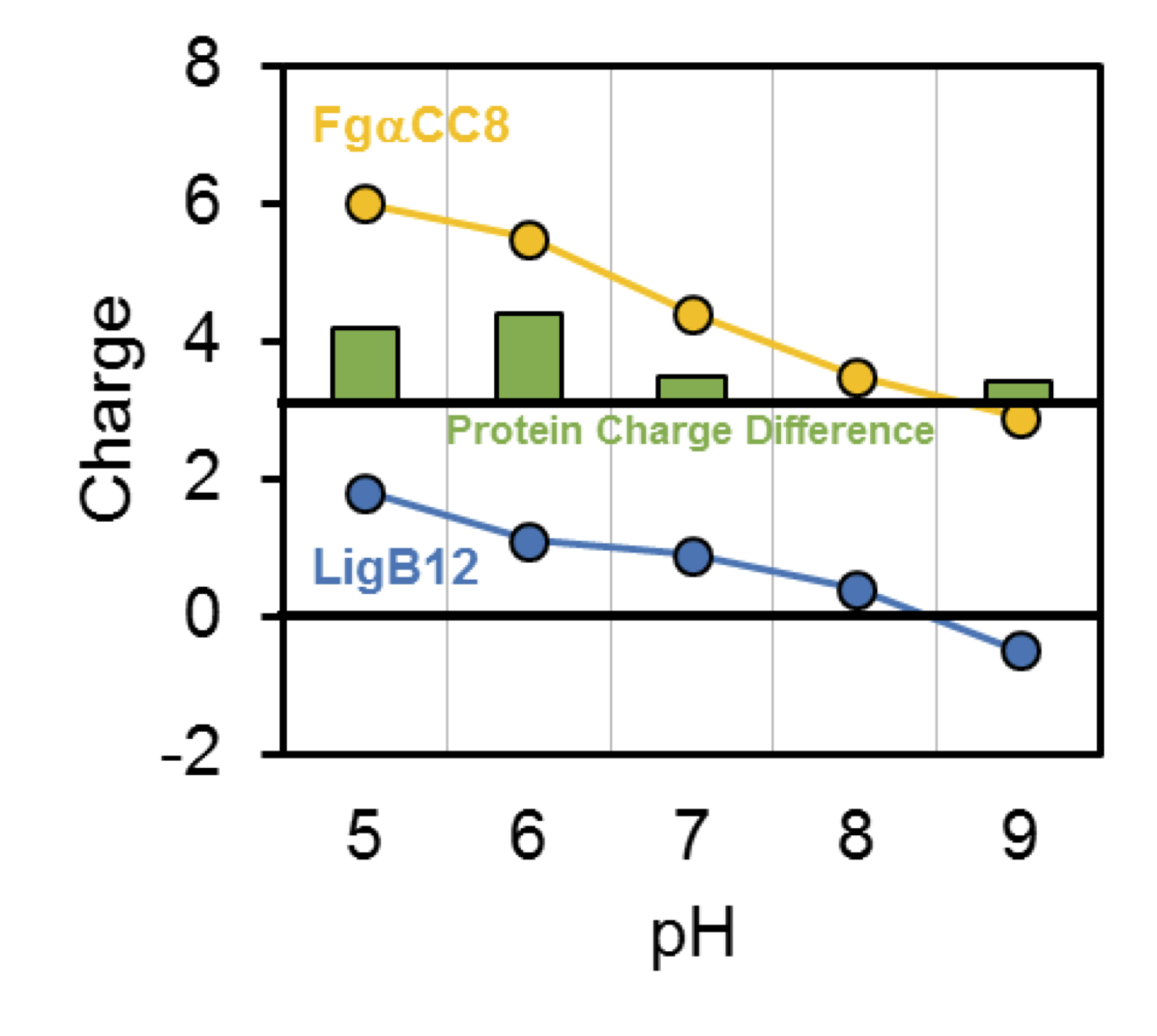

Supplement: S1 Fig — The primary sequence-based charge values for LigB12 and FgαCC8 are plotted for the pH conditions used to assess the pH-dependence of the LigB12-FgαCC8 binding interaction in ELISA studies (Fig 5A). The pH-specific charge for the proteins was calculated using the Protein Calculator version 3.4 (Putnam, C.D., 2013, http://protcalc.sourceforge.net/). The difference between the charge of the LigB12 and FgαCC8 at each pH is shown as a column chart relative to the charge difference at pH 8 (+3.1, the smallest difference). The largest LigB12-FgαCC8 charge difference occurs at pH 6. (TIF) [file pntd.0004974.s001.tif]

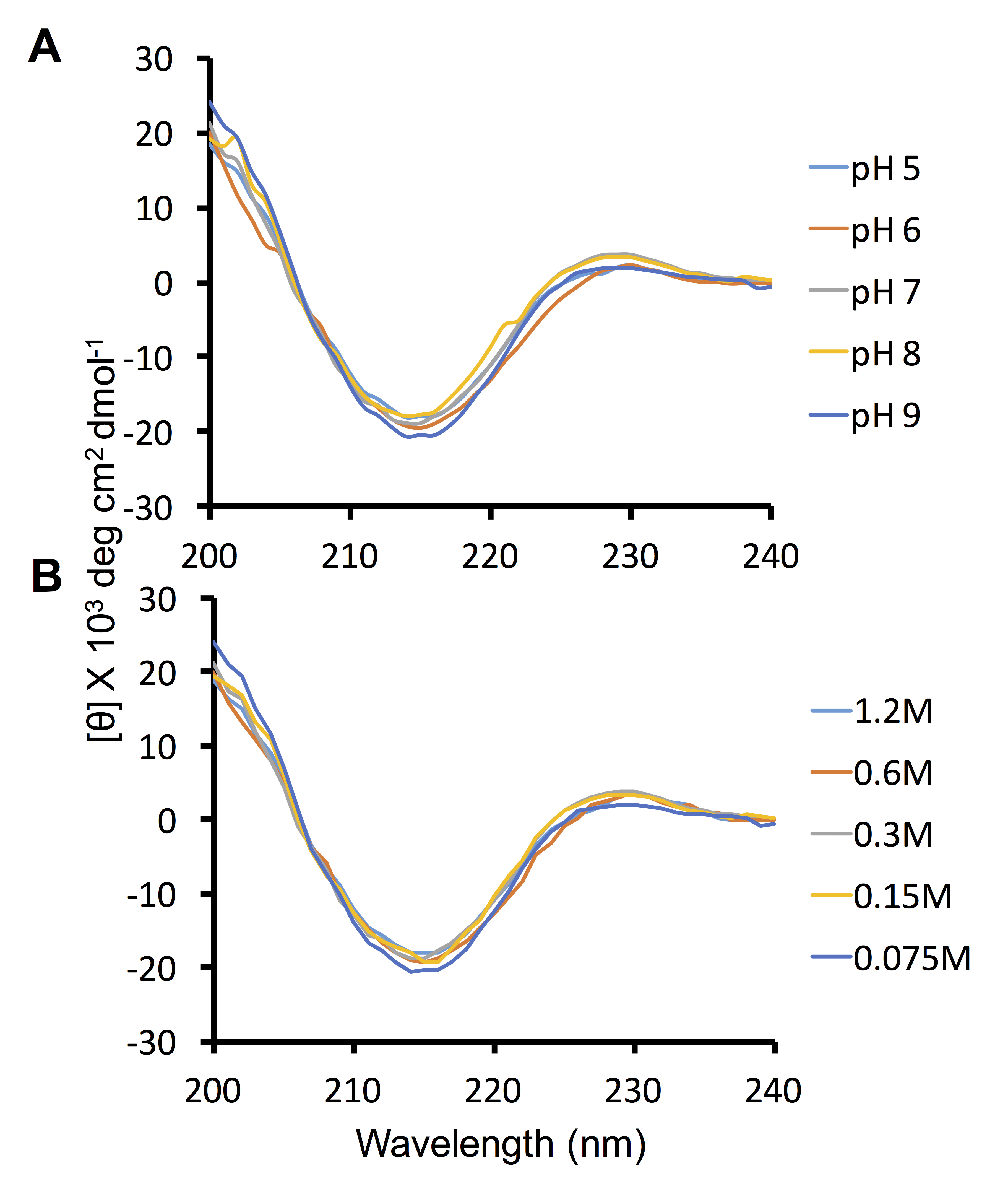

Supplement: S2 Fig — Far-UV circular dichroism analysis of (A) wild-type LigB12 in PBS buffers with different pH and (B) wild-type LigB12 in phosphate buffers with various concentrations of NaCl. The molar ellipticity, θ, was measured from 200 nm to 240 nm for 10μM of each protein at room temperature. (TIF) [file pntd.0004974.s002.tif]

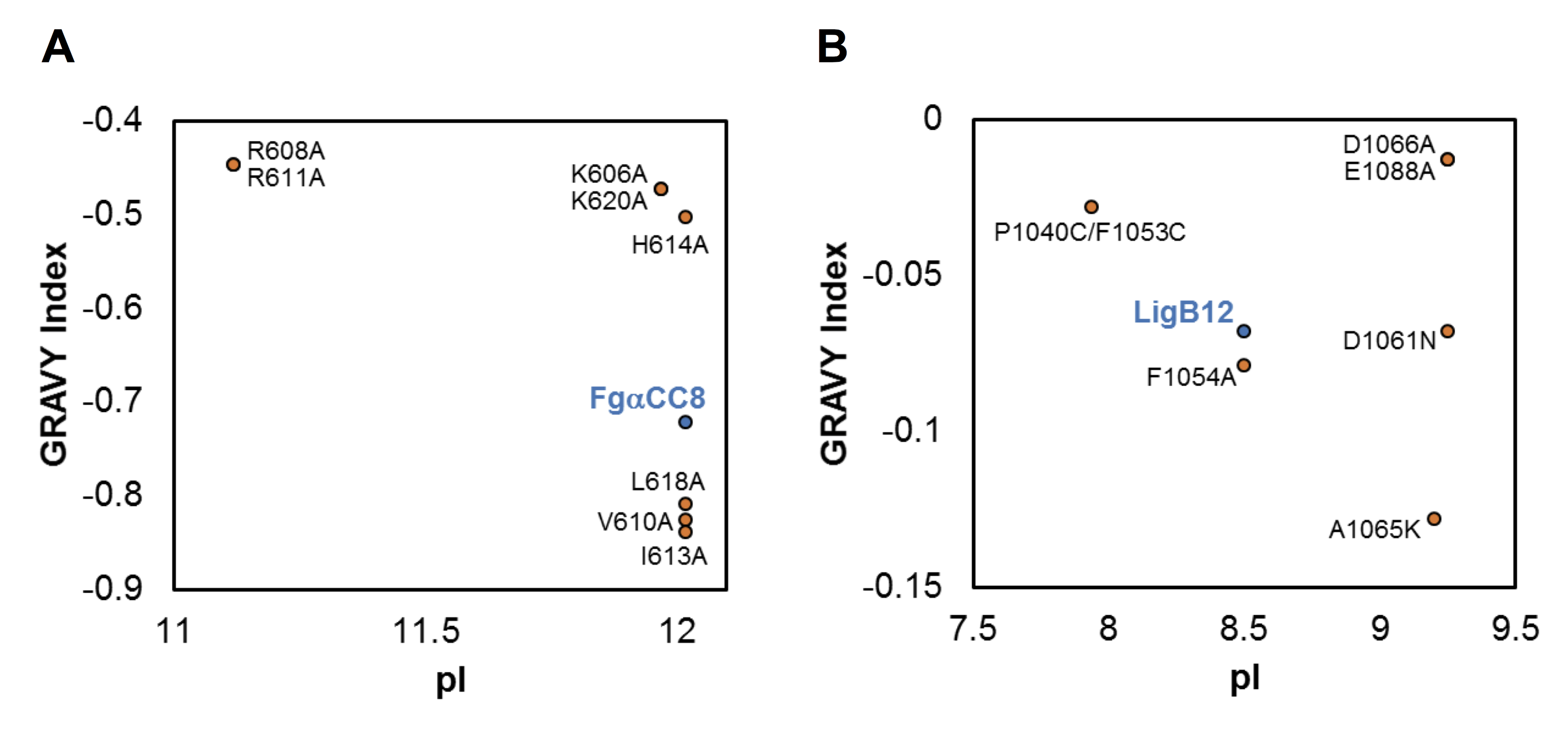

Supplement: S3 Fig — The grand average of hydropathicity (GRAVY) index and theoretical pI are plotted in blue for (A) wild-type FgαCC8 and (B) wild-type LigB12. The position of mutants are also annotated on the plot of GRAVY index vs. pI. The GRAVY index was calculated using the GRAVY Calculator (Fuchs, S., 2011, http://www.gravy-calculator.de/) and the theoretical pI was calculated using the Protein Calculator version 3.4 (Putnam, C.D., 2013, http://protcalc.sourceforge.net/). (TIF) [file pntd.0004974.s003.tif]

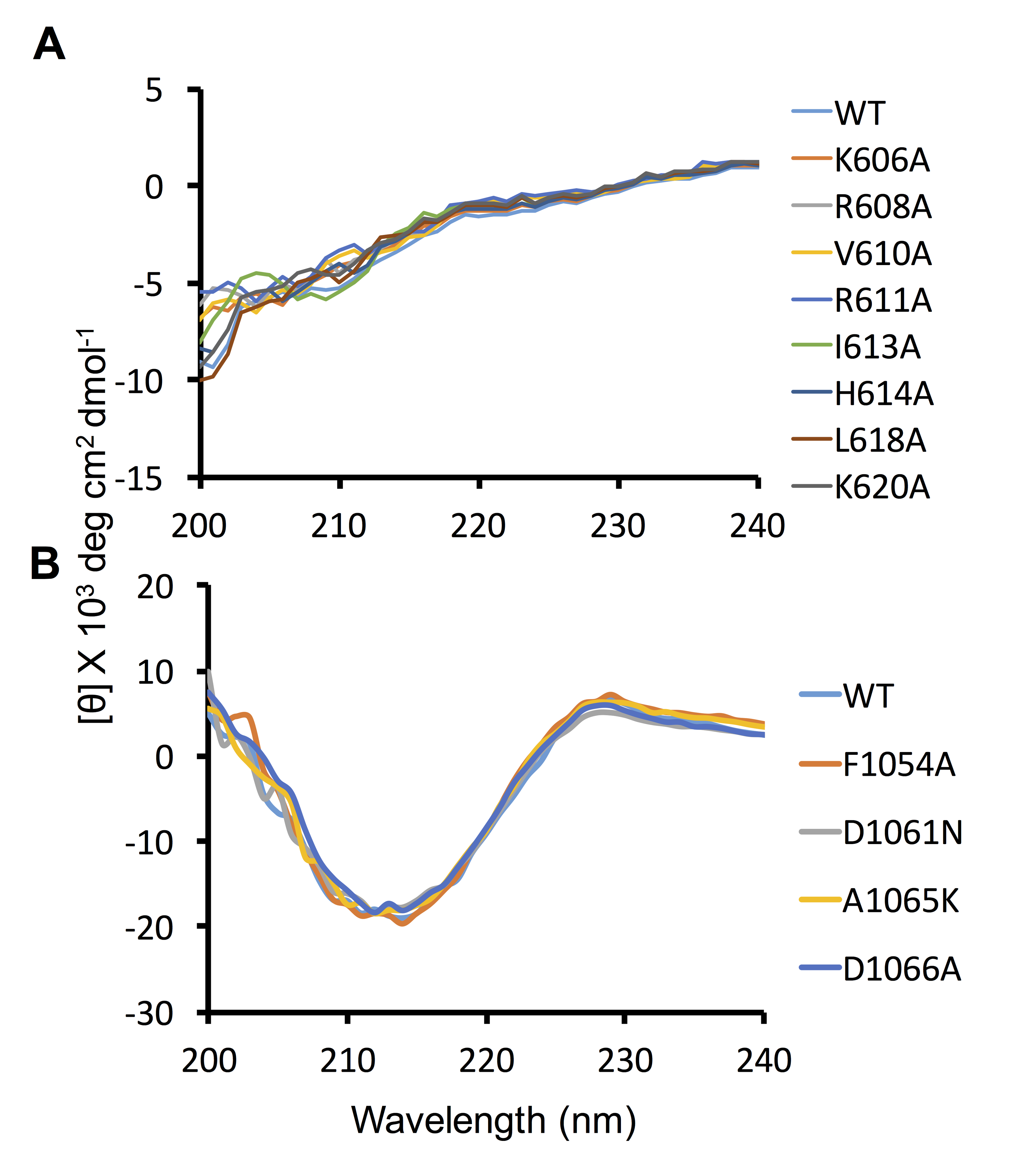

Supplement: S4 Fig — Far-UV circular dichroism analysis of (A) wild-type and mutant FgCC8 in PBS buffer, (B) wild-type and mutant LigB12 in PBS buffer. The molar ellipticity, θ, was measured from 200 nm to 240 nm for 10μM of each protein at room temperature. (TIF) [file pntd.0004974.s004.tif]

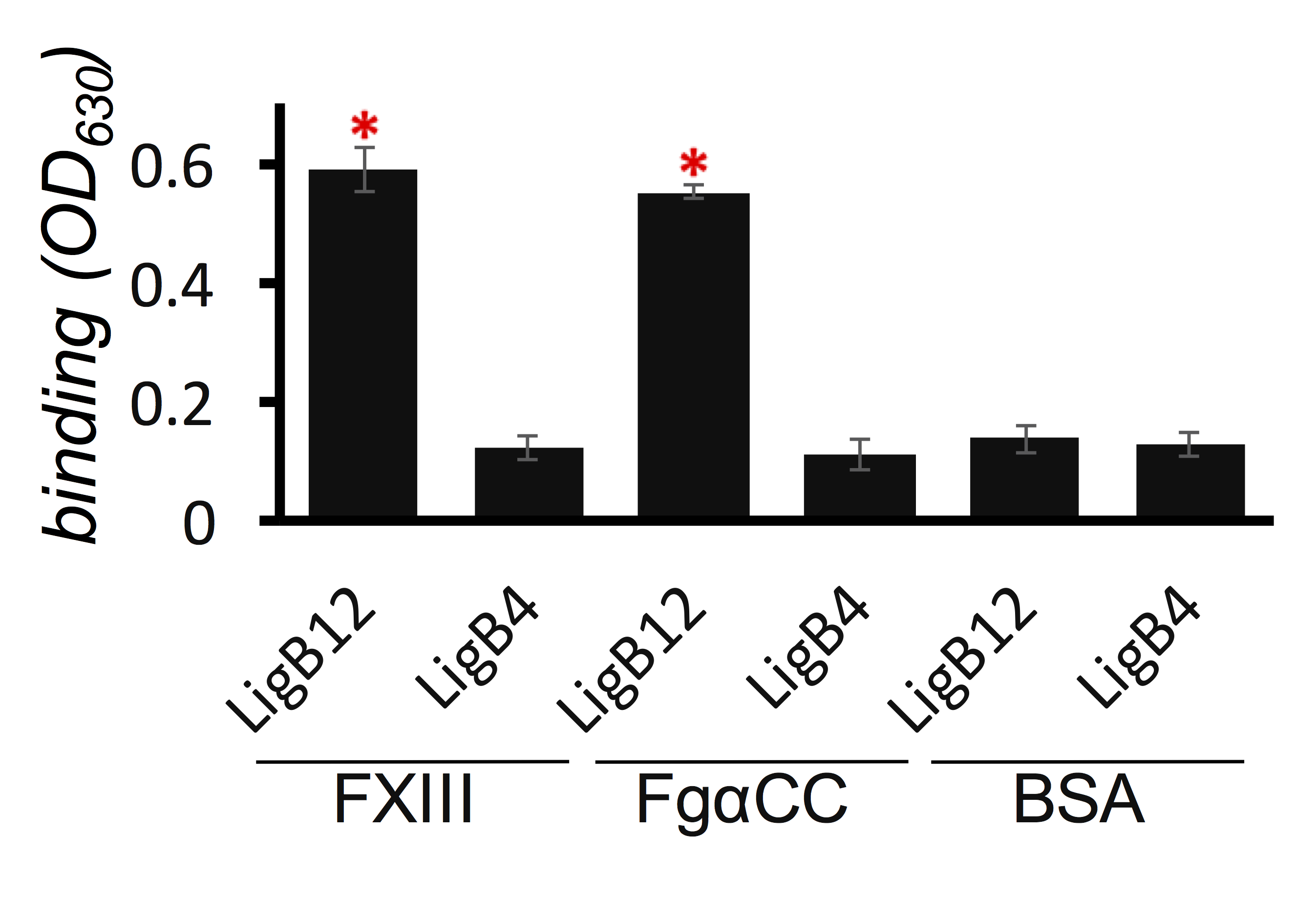

Supplement: S5 Fig — GST tagged LigB12 and LigB4 (3 μM) were added to FXIII, FgαCC and BSA (negative control) immobilized wells. The binding of LigB12 or LigB4 to FXIII or FgαCC was measured by ELISA using HRP-conjugated anti-GST antibodies. The mean +/- 1 standard deviation shown in the graph was derived from three independent experiments. The significant binding of LigB12 to FXIII or FgαCC as opposed to BSA control (ANOVA test, p < 0.05) was marked by an asterisk. LigB4 did not show any significant binding to either FXIII or FgαCC (ANOVA test, p > 0.1). (TIF) [file pntd.0004974.s005.tif]
